# Data on cell cycle in breast cancer cell line, MDA-MB-231 with ferulic acid treatment

**DOI:** 10.1016/j.dib.2016.02.001

**Published:** 2016-02-12

**Authors:** Eunmi Park

**Affiliations:** Department of Food and Nutrition, School of Life Science and Nano-Technology, Hannam University, Daejeon, Republic of Korea

## Abstract

Inhibition to repair DNA metabolism to respond to damaged DNA can lead to genetic instability, resulting in cancer cell death (Audeh et al., 2010; Bryant et al., 2005; Farmer et al., 2005; Lukas et al., 2003; Tutt et al., 2010) [1], [2], [6], [8], [11]. Despite of various studies demonstrating efficiency of combination therapy through down-regulation of DNA repair pathway, the suppression effects of DNA repair pathway by chemotherapeutic agents from natural bioactive compounds are less understood (Eitsuka et al., 2014; Kastan et al., 2004; Kawabata et al., 2000; Mancuso et al., 2014) [5], [7], [9].

Here, the data shows that ferulic acid reduced the S-phases post to UV treatment in breast cancer cells and was hypersensitive in breast cancer cells, MDA-MB-231.

**Specifications Table**

**Table:** 

Subject area	Biology
More specific subject area	Cancer biology
Type of data	Figure, graph
How data was acquired	FACS analysis and colony assay
Data format	Analyzed with FACS data, colony assay and statistical tests
Experimental factors	Comparison of ferulic acid with UV-induced DNA damage in MDA-MB−231 cells
Experimental features	Cell cycle analysis and colony assay in breast cancer cell line, MDA-MB−231 with ferulic acid treatment post to UV.
Data source location	Daejeon, Korea
Data accessibility	*Data is within this article*

**Value of the data**

• The data significantly extends ferulic acid treatment in breast cancer chemotherapy.
• The data provides the information of the effect of different UV irradiations in breast cancer cells with ferulic acid treatment.

## 1 Data

**Fig. 1:**
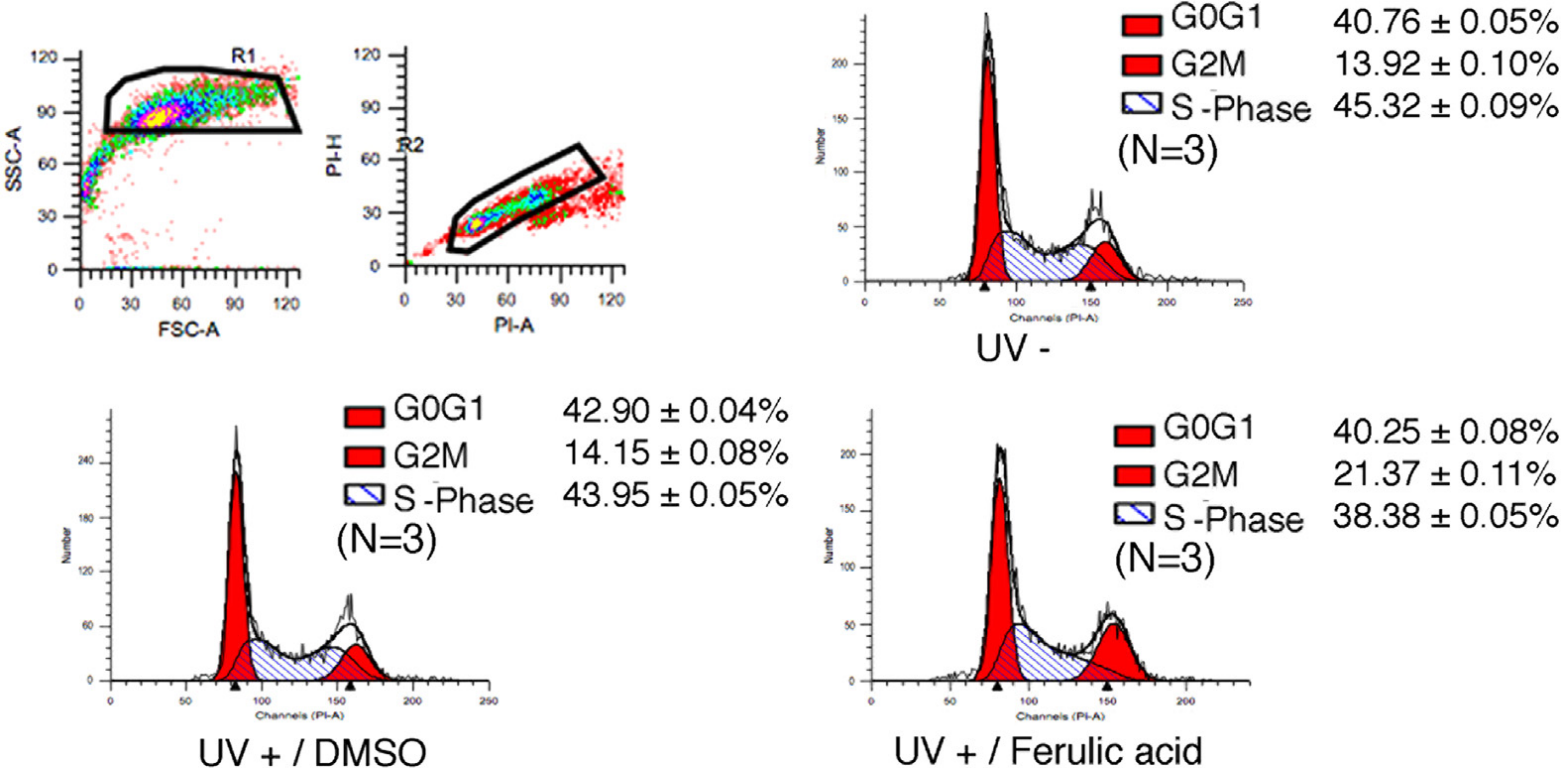
Ferulic acid reduces S-phase cell cycle profiles post to UV treatment. MDA-MB-231 cells were cultured with 10 μM ferulic acid/or DMSO for 24 h. The cells were exposed to UV treatment and harvested. The cell pellets were fixed in 70% ethanol and stained with PI for FACS analysis. UV-; UV untreated, UV+; UV treated (20 mJ/s, harvest post to 3 h).

**Fig. 2:**
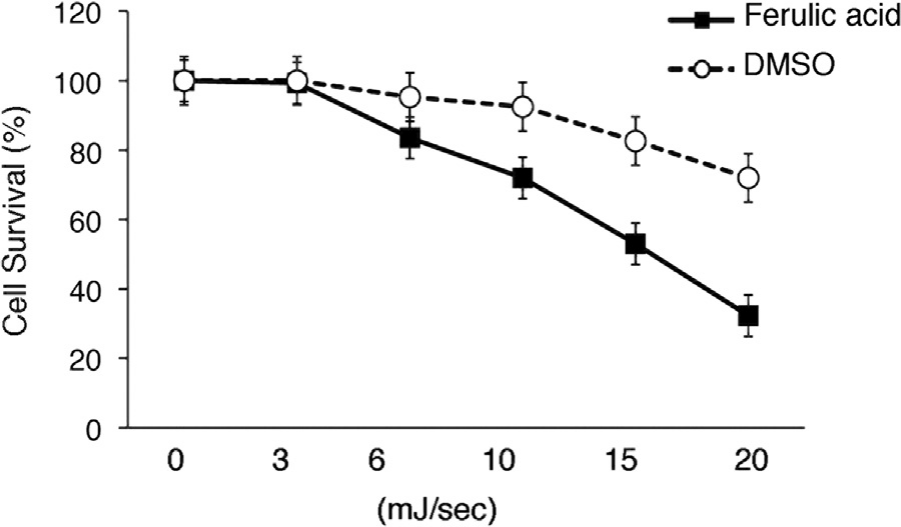
Breast cancer cells with ferulic acid treatment are highly sensitive to UV treatment. MDA-MB-231 cells were pretreated with ferulic acid (10 μM) or DMSO for 24 h and re-plated in culture dishes. Then the cells were exposed with UV irradiation (0−20 mJ/s). Survival was determined using a colony assay from three independent experiments. The data are mean±standard errors. *; *p*<0.05.

FACS profiles showed that ferulic acid in combination with UV irradiation reduced more the S-phase compared to the cells treated with UV irradiation, as well as in UV untreated cells [3] (Fig. 1). MDA-MB-231 cells with ferulic acid were more sensitive to UV treatment compared to the cells with DMSO by performing colony formation assays [4] (See Fig. 2).

## 2 Experimental design, materials and methods

### 2.1 Cell culture

MDA-MB-231 breast cancer cells were cultured in DMEM (Invitrogen) supplemented with 10% fetal bovine serum (Invitrogen) and 1% penicillin/streptomycin (Invitrogen) [3], [4]. The cells were cultured with ferulic acid (Sigma) treatment for experiments. Ferulic acid was dissolved in DMSO was dissolved in PBS for the experiments. All of cell lines were incubated at 37 ^°^C with 5% CO_2_.

### 2.2 Cell cycle analysis

MDA-MB-231 breast cancer cells were pretreated with ferulic acid or DMSO for 24 h. The cells were exposed to 20 mJ/s UV treatment and harvested post to 3 h. For fluorescence-activated cell sorting (FACS) analysis, MDA-MB-231 cells were fixed overnight at 4C in 70% ethanol, stained with propidium iodine (PI) for 1 h. The cells analyzed for DNA content using a FACS Calibur machine (BD Biosciences).

### 2.3 Colony assay (Cell survival analysis)

MDA-MB-231 breast cancer cells were prepared for colony assay [10]. After the pre-treatments with ferulic acid or DMSO, the cells were plated in plates with UV irradiation (0−20 mJ/s). The cells were cultured for clonogenic assay in triplicates. After 2 weeks in culture, colonies were fixed with methanol and stained with crystal violet.

### 2.4 Statistical analysis

All data are representative of at least three independent experiments. Data are mean±SEM unless otherwise indicated. Statistical significance of comparison between two groups was determined by two-tailed Student׳s *t*-test where indicated. For comparing more than one group, one-way ANOVA was used. Significant differences were considered at *p*-values of less than 0.05.
